# Inhibition of DRP-1-Dependent Mitophagy Promotes Cochlea Hair Cell Senescence and Exacerbates Age-Related Hearing Loss

**DOI:** 10.3389/fncel.2019.00550

**Published:** 2019-12-17

**Authors:** Hanqing Lin, Hao Xiong, Zhongwu Su, Jiaqi Pang, Lan Lai, Huasong Zhang, Bingquan Jian, Weijian Zhang, Yiqing Zheng

**Affiliations:** ^1^Department of Otolaryngology, Sun Yat-sen Memorial Hospital, Sun Yat-sen University, Guangzhou, China; ^2^Institute of Hearing and Speech-Language Science, Sun Yat-sen University, Guangzhou, China

**Keywords:** presbycusis, oxidative stress, DRP-1, mitophagy, cochlea

## Abstract

**Background**: Mitochondrial dysfunction is considered to contribute to the development of age-related hearing loss (AHL). The regulation of mitochondrial function requires mitochondrial quality control, which includes mitophagy and dynamics. Dynamin-related Protein 1 (DRP-1) is believed to play a central role in this regulation. However, the underlying mechanism of DRP-1 in AHL remains unclear. Here, we examined whether the decline of DRP-1-dependent mitophagy contributes to the development of AHL.

**Methods**: We induced cellular and cochlear senescence using hydrogen peroxide (H_2_O_2_) and evaluated the level of senescence through senescence-associated β-galactosidase staining. We evaluated mitophagy levels *via* fluorescence imaging and Western Blotting of LC3II and P62. Mitochondrial function was assessed by ATP assay, mtDNA assay, and JC-1.

**Results**: We found that both the expression of DRP-1 and the mitophagy level decreased in senescent cells and aged mice. DRP-1 overexpression in HEI-OC1 cells initiated mitophagy and preserved mitochondrial function when exposed to H_2_O_2_, while cells with DRP-1 silencing displayed otherwise. Moreover, inhibition of DRP-1 by Mdivi-1 blocked mitophagy and exacerbated hearing loss in aged C57BL/6 mice.

**Conclusion**: These results indicated that DRP-1 initiated mitophagy, eliminated mitochondrial dysfunction, and may protect against oxidative stress-induced senescence. These results provide a potential therapeutic target for AHL.

## Introduction

Aging is a physiological phenomenon that occurs in all eukaryotes. Age-related hearing loss (AHL) is one of the most serious health-related social problems (Roth et al., 2011). It is characterized by an age-dependent decline of auditory function that is attributable to the loss and dysfunction of hair cells and spiral ganglion cells in the cochlea of the inner ear (Lin et al., 2013). There is agreement that cumulative mitochondrial dysfunction and Reactive Oxygen Species (ROS) exacerbate hearing loss. Still, the exact molecular mechanism of AHL remains unknown. A series of cellular activities are regulated in the cellular aging process. Among these, mitochondrial function is considered to be a central event in various diseases in all systems and at all ages. A recent study shows that mitochondrial dysfunction is one of the characteristics of the aging process (López-Otín et al., 2013), and it is thought to induce diseases such as Alzheimer’s (Lee et al., 2010) and AHL (Seo et al., 2017).

Cellular activities require mitochondria to provide energy, but meanwhile, adenosine triphosphate (ATP) synthesis oxidative stress is also produced, causing injury to cells and organelles. Excessive oxidative stress and damaged organelles can be eliminated and recycled by an essential process called autophagy (Mizushima et al., 2008). Specific mitochondrial autophagy is called mitophagy and allows dysfunctional mitochondria to go through a process of degradation. Mitophagy, along with mitochondrial biogenesis, maintains mitochondrial function.

Mitochondria constantly undergo fission and fusion to realize mitochondrial quality control. The activation of dynamin-related protein-1 (DRP-1) is required when mitochondria go through fission to create new mitochondria or to separate damaged mitochondria matrices so as to preserve normal mitochondrial function during high levels of cellular stress (Youle and van der Bliek, 2012). The main approach to eliminate injured mitochondria by autophagy is mitophagy. Disturbance of mitophagy may affect normal mitochondrial function and the physiological or pathological cellular condition. DRP-1 is a highly conserved GTPase that plays the core role in mitophagy by regulating the separation of mitochondria (Yoneda et al., 1994). When mitochondrial fission occurs, DRP-1 is recruited from the cytosol to the outer membrane, where it forms a spiral complex around the mitochondria (Ingerman et al., 2005). One situation is symmetrical replicative fission. This is where a healthy mitochondrion divides equally into two daughter mitochondria, which generates new mitochondria by mitochondrial biogenesis or by fusion (Gray et al., 1999). The other situation is where asymmetrical fission is initiated so that damaged mitochondria can be removed through mitophagy (Yang and Yang, 2013). Dysfunctional mitochondria display relatively depolarized membrane potential, which can be targeted for selective autophagy for further elimination.

In our study, we hypothesized that the expression and normal functions of DRP-1 and DRP-1-dependent mitophagy are disrupted in the aged cochlea with AHL. This disruption is considered harmful to the survival and function of cochlear hair cells. To determine whether DRP-1 and mitophagy play a role in aged cochlea with AHL, we examined DRP-1 and mitophagy and their roles in cellular senescence and AHL.

## Materials and Methods

### Animals

C57BL/6 mice were purchased from the Laboratory Animal Center, Sun Yat-sen University (Guangzhou, China). C57BL/6 mice were randomly divided into three groups: one “young control” group (1 month old), one “old control” group (12 months old), one “old Mdivi-1 IP” group (12 months old). Mdivi-1 was administrated intraperitoneally in the amount of 12 mg/kg every 3 days. All experiments were performed according to protocols approved by the Animal Research Center, Sun Yat-sen University.

### Auditory Brainstem Response

Auditory brainstem response (ABR) tests were conducted using the Tucker-Davis Technology (TDT) System III (Alachua, FL, USA) as previously described (Pang et al., 2017). In brief, mice were anesthetized, and subdermal needle electrodes were inserted at the vertex (active) and below the left ear (reference) and the right ear (ground). ABR tests were measured at 8, 16, and 32 kHz. The average response to 1,024 stimuli was obtained through reduction of the sound intensity in 5 dB intervals near the threshold, which was defined as the lowest stimulus level where a positive wave was evident.

### Tissue Preparation

C57BL/6 mice were decapitated after the ABR tests. The temporal bones were dissected, and the cochleae were obtained and fixed with 4% paraformaldehyde at 4°C overnight and decalcified in 4% sodium ethylenediaminetetraacetic acid for 2 days. For protein preparation, cochlear basilar membranes were dissected from cochlea, snap-frozen in liquid nitrogen, and stored at −80°C.

### Cell Culture

Cochlear HEI-OC1 cells (kindly provided by F. Kalinec from the House Ear Institute, Los Angeles, CA, USA) were cultured in Dulbecco’s Modified Eagle’s Medium (Gibco, USA) and supplemented with 10% fetal bovine serum (Gibco) at 33°C under a 10% CO_2_ condition.

### Primary Tissue Culture

P2–3 C57BL/6 mice were purchased from Guangdong Medical Laboratory Animal Center (GDMLAC). The temporal bones were dissected, and the cochleae were obtained and then supplemented with 10% fetal bovine serum (Gibco) at 37°C under a 5% CO_2_ condition.

### Cell Transfection

DRP-1 Silencer (Ruibo, Guangzhou, China), a commercial mixture of siRNA and ASO (Antisense oligonucleotides), was used to knock down the expression of DRP-1 in HEI-OC1 cells. Cell transfections were performed using Lipofectamine RNAiMAX Transfection Reagent (Life Technologies, USA) following the manufacturer’s instructions. The HEI-OC1 cells were transfected with lentivirus-mediated green fluorescent protein (GFP)-LC3 to generate GFP-LC3-expressing cells. The lentivirus containing the GFP-LC3 fusion gene was purchased from Hanbio (Shanghai, China). HEI-OC1 cells were seeded into six-well dishes (1 × 10^5^ per well) and infected with the recombinant lentivirus. After culturing in the presence of puromycin for 2 weeks, HEI-OC1 cells with GFP-LC3 were selected.

### Western Blot Analysis

Western Blotting was conducted as previously described (Pang et al., 2017). Briefly, proteins were extracted from cells and cochlea tissues using radioimmunoprecipitation assay lysis buffer (Thermo plus, USA). Protein samples (20 μg) were resolved on a 10% SDS-polyacrylamide gel and transferred onto polyvinylidene fluoride membranes (Millipore, Burlington, MA, USA). After being blocked with 5% nonfat milk, the membranes were incubated with anti-DRP1 (1:1,000, Proteintech, Rosemont, IL, USA), anti-p-DRP1 (1:1,000, Abclonal, Woburn, MA, USA), anti-LC3I/II (1:1,000, CST, USA), anti-P62 (1:1,000, CST, USA), anti-P53 (1:1,000, Abclonal, Woburn, MA, USA), and anti-P21 (1:1,000, Abclonal, Woburn, MA, USA) at 4°C overnight, followed by incubation with secondary antibodies (1:3,000) at room temperature for 1 h. The immunoreactive bands were then detected by enhanced chemiluminescence (Millipore, Burlington, MA, USA). Band intensities were analyzed using ImageJ (NIH, USA). β-actin was applied as loading and internal control.

### Cell Viability Assay

Cell viability was detected using CCK-8 kits (DOJINDO, Japan) according to the manufacturer’s protocols. Briefly, cells were plated into 96-well plate. At the indicated time after treatments, 10 μl of CCK-8 solution was added to the wells. Cells were then incubated at 37°C for 1 h. Finally, a microplate reader (Labsystems Dragon, Finland) was used to measure the absorbance at 450 nm.

### Cell Population Doubling Rate

For the population doubling rate assay, cells were counted, and 1 × 10^5^ cells were plated in every 35 mm dish, pre-treated, replaced with a normal culture medium, and then incubated under permissive conditions. Cells were harvested and counted and re-planted every 72 h. The formula below was used to evaluate the population doubling rate.

f(x)=(log⁡N1−log⁡N2)/log⁡2

(N1 = Number of cells counted, N2 = Number of cells plated)

### Senescence-Associated β-Galactosidase Stain

Cellular senescence-associated β-galactosidase staining was conducted using a senescence β-galactosidase staining kit (Beyotime, Shanghai, China) following the manufacturer’s instruction. Briefly, cells were plated in 6-well plate and pre-treated. Cells were gently washed with PBS and fixed with fixing solution. After being washed with PBS three times, 1 ml of staining solution was added to each well, which was then sealed with parafilm and incubated at 37°C without CO_2_ overnight.

### ATP Assay

ATP assay was performed using an ATP Assay Kit (Beyotime, Shanghai, China) following the manufacturer’s instructions. Briefly, cells were homogenized with lysis buffer and centrifuged at 12,000 *g* for 5 min at 4°C. An ATP detection reagent was diluted with dilution buffer and added to 96-wells. Then, the samples were added into the wells and mixed with the detection solution. The chemiluminescence intensities of samples and standards were measured with a SpectraMax M5 microplate reader (Molecular Devices, San Jose, CA, USA). The levels of ATP were calculated based on the standard curve and normalized to the protein content.

### Mitochondrial Fluorescent Probe Staining Analysis

Mitochondrial staining was conducted with the mitochondrial probe MitoTracker Red CMXRos (Yeasen, Shanghai, China) according to the manufacturer’s protocols. After being washed with PBS, the cells were counterstained with DAPI for 10 min and imaged with an Olympus BX63 microscope (Olympus, Japan).

### Mitochondrial DNA (mtDNA) Content Analysis

Total genomic DNA was extracted from cells using a Universal Genomic DNA Extraction Kit (Takara) according to the manufacturer’s protocols. The mtDNA levels were quantified by qPCR on a Roche LightCycler 96 (Roche) using D-loop primers (forward: 5′-GGTTCTTACTTCAGGGCCATCA-3′, reverse: 5′-GATTAGACCCGTTACCATCGAGAT-3′). Nuclear gene beta2-microglobulin (B2M) primers (forward: 5′-ATGGGAAGCCGAACATACTG-3′, reverse: 5′-CAGTCTCAGTGGGGGTGAAT-3′) were used as a nuclear control.

### Statistical Analysis

All experiments were independently repeated at least three times. Data were presented as mean ± SD and were analyzed with SPSS and Graphpad Prism 5 software. Student’s *t*-test and one-way ANOVA were used for statistical analysis. Values with *p* < 0.05 were considered significant.

## Results

### Oxidative Stress-Induced Senescence in HEI-OC1 Cells

**Figure 1:**
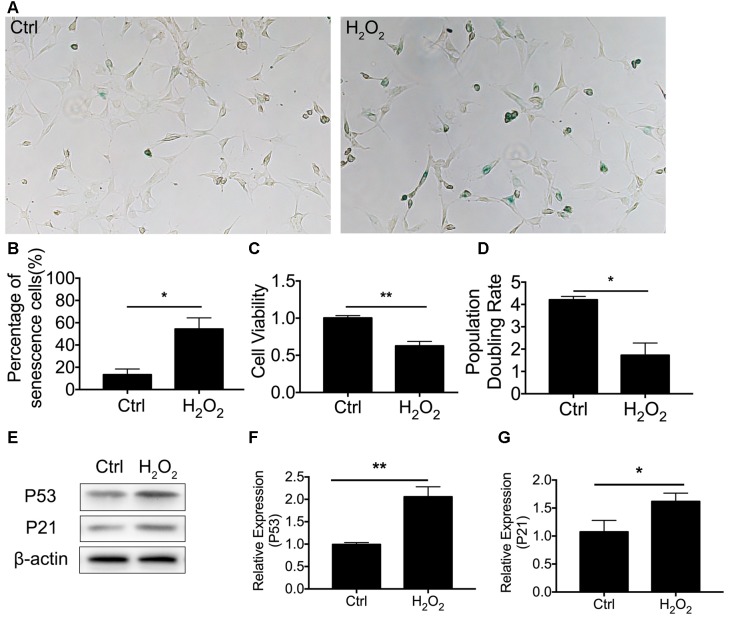
H_2_O_2_-induced cellular senescence in HEI-OC1 cells. **(A)** β-gal staining of senescent HEI-OC1 cells treated with H_2_O_2_. **(B)** Percentage of β-gal stained cells. **(C)** Cell viability of 1 mM H_2_O_2_ treated cells compared with control cells. **(D)** Population doubling rate in HEI-OC1 cells. **(E–G)** Representative Western Blot analysis using antibodies against P53 and P21 to assess cellular senescence. *p < 0.05, **p < 0.01.

We first established cellular senescence by inducing oxidative stress. HEI-OC1 cells were briefly exposed to H_2_O_2_ (1 mM for 1 h), and we then further investigated the cellular molecular change between mitophagy and senescence. Our results revealed that cellular senescence was induced 24 h after H_2_O_2_ treatment at a rate of 54.4 ± 9.94% HEI-OC1 cells stained with β-gal staining (Figure 1A). In the meantime, there was 13.4 ± 2.25% of senescent β-gal-stained cells in the normal control HEI-OC1 cells (*p* < 0.0001, Figure 1B). We further assessed cellular senescence with cell viability, population doubling rate, and senescence-associated P53 and P21. Lower cell viability was detected in cells treated with H_2_O_2,_ being 0.63 ± 0.03-fold lower than the control cells (*p* = 0.0006, Figure 1C). The population doubling rate was calculated to evaluate the aging pattern. Higher rates indicate a higher speed of cell growth. The population doubling rate dropped to 1.73 ± 0.27 compared to normal cells at 4.21 ± 0.08 (*p* = 0.0001, Figure 1D). Cellular senescence-associated P53 and P21 were further assessed by Western Blotting. H_2_O_2_ treatment of HEI-OC1 cells significantly elevated the expression of P53 and P21 (Figures 1E–G). These data demonstrated that H_2_O_2_ induced cellular senescence in HEI-OC1 cochlear cells.

### Oxidative Stress Downregulated the Mitophagy Level and Induced Mitochondrial Dysfunction in Cellular Senescence

**Figure 2:**
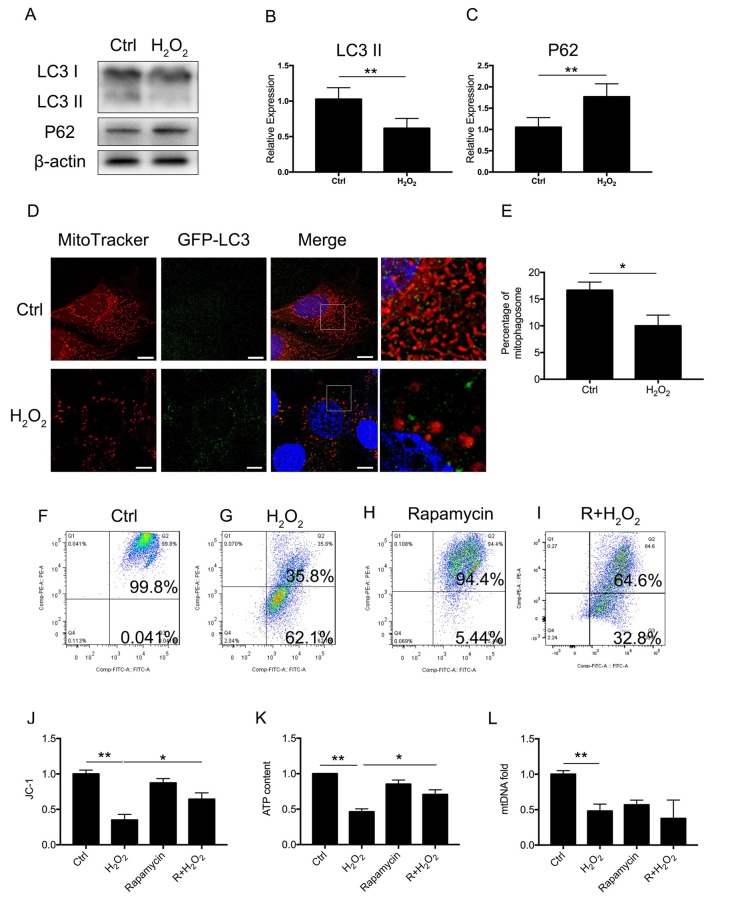
Mitophagy and mitochondrial function of HEI-OC1 cells. **(A)** Representative Western Blot analysis using antibodies against LC3 I/II and P62 to assess autophagy flux. **(B,C)** Relative expression of mitophagy proteins LC3 II and P62 between control cells and H_2_O_2_-treated cells. **(D)** Immunofluorescence image showing mitophagy of mitochondria and autophagosome in green fluorescent protein (GFP)-LC3-expressing HEI-OC1 cells. **(E)** Percentage of mitophagosome in GFP-LC3-expressing HEI-OC1 cells between control and H_2_O_2_ treatment.** (F–I)** Cellular mitochondrial membrane potential between control and H_2_O_2_ treatment according to JC-1 staining and measured by flow cytometry. **(J)** Mitochondrial membrane potential analysis. Rapamycin-initiated autophagy improved mitochondrial membrane potential under H_2_O_2_ treatment. **(K)** ATP content in cells treated with H_2_O_2_ or rapamycin. ATP content was largely preserved with rapamycin under H_2_O_2_ treatment. **(L)** Relative mtDNA fold change, representing relative mitochondria amount. No significant difference between H_2_O_2_ treatment and rapamycin treatment. *p < 0.05, **p < 0.01. Scale bar = 10 μm.

To assess whether there was a molecular change between mitophagy and senescence in HEI-OC1 cells, we further examined blockage of the autophagy flux (Figure 2A). Western Blotting revealed that 1 mM of H_2_O_2_ treatment resulted in a decrease of LC3 II of 0.62 ± 0.08-fold relative to control and a 1.77 ± 0.18-fold relative increase of P62 (*p* < 0.05, Figures 2B,C). The Western Blotting results revealed the suppression of the autophagy function. To further determine mitophagy, we used transfected HEI-OC1 cells expressing GFP-LC3 and staining with the MitoTracker Red fluorescence probe. The yellow puncta displayed were considered as the merging of mitochondria and autophagosomes (Figure 2D). From each group, 20 random cells were counted. The percentage of mitophagosome with H_2_O_2_ treatment was 10 ± 1.16% compared to the control group, with 16.67 ± 0.88% (*p* = 0.0101, Figure 2E). The data indicated that mitophagy was suppressed in H_2_O_2_-induced senescent HEI-OC1 cells.

Next, we examined whether mitophagy is beneficial to mitochondrial function. We treated the cells with 0.5 μM rapamycin for 8 h to initiate autophagy and mitophagy. To evaluate mitochondrial dysfunction induced by oxidative stress and to determine if mitophagy would rescue mitochondrial function, we evaluated mitochondrial membrane potential by JC-1, ATP content, and mtDNA in HEI-OC1 cells treated with H_2_O_2_. The mitochondrial membrane potential of each group was evaluated with JC-1 stain by flow cytometry. Cells in the H_2_O_2_ treatment group displayed a lower percentage of mitochondria with normal potential at a rate of 39.91 ± 0.04% (*p* < 0.0001, Figures 2F,G,J). We found that treatment with rapamycin under oxidative stress increased the percentage of mitochondria with normal potential compared to H_2_O_2_ treatment alone (*p* = 0.0152, Figures 2H,I). Also, we examined the ATP content under H_2_O_2_ treatment and also with rapamycin. H_2_O_2_ decreased the ATP content of HEI-OC1 cells to ~0.46 ± 0.02-fold that of the control group, while, with pre-treatment with rapamycin, H_2_O_2_ only resulted in a decline of ATP content to ~0.71 ± 0.04 fold (*p* = 0.0058, Figure 2K). However, there is no significant difference between H_2_O_2_ and rapamycin treatment (*p*_(H2O2, rapamycin)_ = 0.74, *p*_(H2O2, R + H2O2)_ = 0.28, Figure 2L) in the amount of mtDNA. These results suggested that H_2_O_2_ suppressed autophagy and mitophagy and resulted in mitochondrial dysfunction.

### DRP-1 Contributed to Mitophagy and Mitochondrial Function in HEI-OC1 Cells Under Oxidative Stress

**Figure 3:**
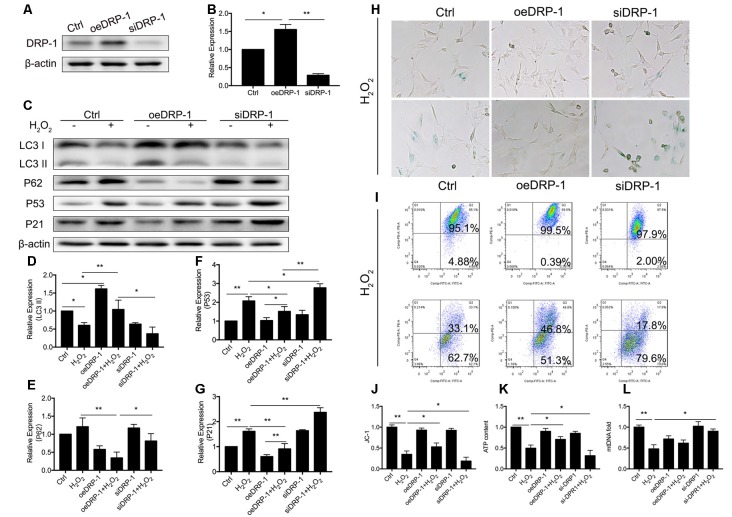
Regulation of Dynamin-related Protein 1 (DRP-1) in cellular senescence and mitochondrial function. **(A)** Representative Western Blot analysis using antibodies against DRP-1 to assess DRP-1 expression. **(B)** DRP-1 expression in overexpressing and silencing HEI-OC1 cells. Relative expression of DRP-1 in overexpressing and silencing HEI-OC1 cells compared to control.** (C)** Representative Western Blot analysis using antibodies against LC3 I/II, P62, P53, and P21 to assess the mitophagy level and cellular senescence in HEI-OC1 cells. **(D,E)** Relative expression of LC3 II and P62. H_2_O_2_ down-regulated mitophagy in all groups. The relative mitophagy was higher in DRP-1 overexpressing cells compared to the control. The mitophagy level was down-regulated in DRP-1 silencing cells. **(F,G)** Relative expression of P53 and P21. H_2_O_2_ induced cellular senescence in HEI-OC1 cells, which elevated the expression of P53 and P21. P53 and P21 expression were relatively higher in DRP-1 silencing cells than in DRP-1 overexpressing cells. **(H)** Senescent HEI-OC1 cells with DRP-1 overexpression and silencing, assessed by β-Gal stain. **(I,J)** Mitochondrial membrane potential, evaluated by FCM with JC-1 staining. **(K)** ATP content in cells overexpressing and silencing DRP-1 under H_2_O_2_ treatment. **(L)** The amount of mitochondria were evaluated by mtDNA measurement. *p < 0.05, **p < 0.01.

DRP-1 plays the core role in mitophagy through regulation of mitochondrial fission. Mitochondrial fission separates injured mitochondria, and this is followed by mitophagy. With a link between declining levels of mitophagy and aging, we hypothesized that the accumulation of damaged mitochondria in senescent cells was due to suppressed mitochondrial fission and subsequent mitophagy. To investigate whether DRP-1 regulates mitophagy, we assessed mitophagy in DRP-1 overexpressing and silencing cells (Figures 3A,B). Autophagy flux and cellular senescence were assessed by Western Blotting of LC3II, P62, P53, and P21 (Figure 3C). Assessment *via* LC3 II and P62 revealed that autophagy flux was blocked in DRP-1 silencing cells but not in DRP-1 overexpressing cells (*p* < 0.01, Figures 3D,E). We further assessed cellular senescence in DRP-1 overexpressing and silencing cells under H_2_O_2_ treatment. We evaluated the expression of senescence-associated P53, P21 (Figures 3F,G), and β-Gal stain (Figure 3H). The elevation of P53 and P21 expression and an increased portion of β-Gal stained cells revealed that silencing DRP-1 suppressed resistance to H_2_O_2_-induced cellular senescence. We then evaluated mitochondrial function in DRP-1 overexpressing and silencing cells to further understand mitophagy levels. DRP-1 overexpressing or silencing in HEI-OC1 cells resulted in no difference in the percentage of mitochondrial membrane potential compared with control cells. However, when cells faced oxidative stress, DRP-1 overexpression rescued mitochondrial membrane potential, displaying 53.0 ± 0.05% of healthy mitochondria and DRP-1 silencing of 18.8 ± 0.05% (*p* < 0.05, Figures 3I,J). The ATP content in DRP-1 overexpressing cells decreased but remained at 0.71 ± 0.04-fold that of the control group. The mtDNA remained at 0.62 ± 0.04-fold that of the control in DRP-1 overexpressing cells treated with H_2_O_2_ (*p* < 0.05, Figures 3K,L). In contrast, cells silencing DRP-1 displayed a significant decrease in ATP content to 0.32 ± 0.07-fold that of the control but with mtDNA at 0.90 ± 0.03 that of the control (*p* < 0.05, Figures 3H,I). Together with the blockage of autophagy flux, we may assume that mitophagy in HEI-OC1 cells was also blocked. The data revealed that DRP-1 overexpression initiated mitophagy and that such cells retained most of the mitochondrial function under oxidative treatment, while silencing DRP-1 inhibited mitophagy. Cells retained most of the mitochondria but less ATP content, indicating that mitochondrial function was disturbed in DRP-1 silencing cells under oxidative stress.

### Increase of Oxidative Stress Downregulated DRP-1 Expression and Resulted in Mitochondrial Dysfunction in Cochlea

**Figure 4:**
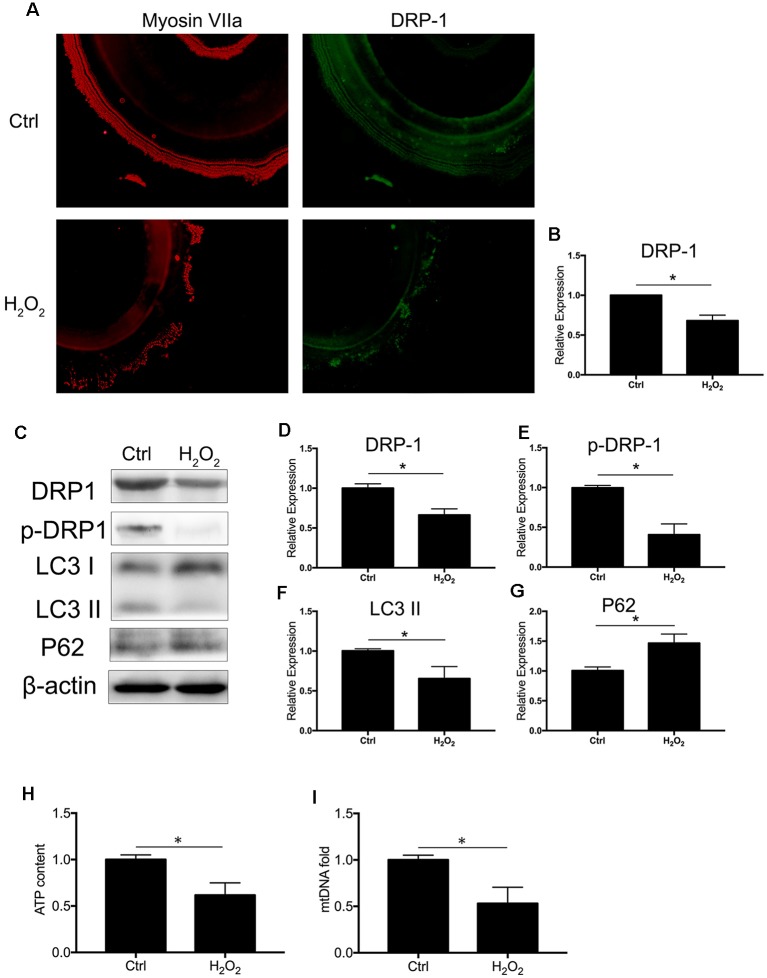
H_2_O_2_ inhibited DRP-1 and mitophagy in cochlea. **(A)** Representative image of immunofluorescence staining of DRP-1 in cochlear explant. **(B)** Down-regulated expression of DRP-1 in cochlea treated with H_2_O_2_.** (C)** Representative Western Blot analysis using antibodies against DRP-1, p-DRP-1, LC3 I/II, and P62 to assess the mitophagy level in cochlear explant. **(D,E)** Relative expressions of DRP-1 and p-DRP-1 in H_2_O_2_-treated cochlea. **(F,G)** Relative expression of LC3 II and P62 in H_2_O_2_-treated cochlea. **(H,I)** Mitochondrial function evaluated by ATP content and mtDNA. H_2_O_2_-treated cochlea revealed a low ATP content and mitochondrial amount. *p < 0.05. Scale bar = 300 μm.

We observed the regulation of DRP-1 in mitochondrial functions and further investigated DRP-1 expression patterns in cochlear explants. Senescence was induced by 0.1 mM H_2_O_2_ treatment in the cochlear explants. We found a significant decrease of DRP-1 (0.66 ± 0.04-fold of control, *p* = 0.0017) and s616 phosphorylated DRP-1 (p-DRP1, 0.41 ± 0.08-fold of control, *p* = 0.0017, Figures 4A–E) in the cochlear explants. The mitophagy level of the cochlear explants was determined by the level of autophagy flux and mitochondrial function. Western Blotting showed the LC3 II and P62 protein levels to decrease (*p*_LC3 II_ = 0.0164, *p*_P62_ = 0.0062), suggesting that autophagy flux was also blocked in H_2_O_2_-treated cochlear explants (*p* = 0.0188, Figures 4F,G). H_2_O_2_ also resulted in mitochondrial dysfunction in the cochlear explants by down-regulating the ATP content to 0.62 ± 0.08-fold (*p* = 0.0095) and mtDNA to 0.53 ± 0.09-fold (*p* = 0.0071) that of control (Figures 4H,I). These results indicated that H_2_O_2_ treatment induced DRP-1 down-regulation and the injury of mitophagy in cochlear explants.

### Fission Inhibitor Mdivi-1 Inhibited DRP-1-Mediated Mitophagy and Exacerbated Senescence in Cochlea Under Oxidative Stress

**Figure 5:**
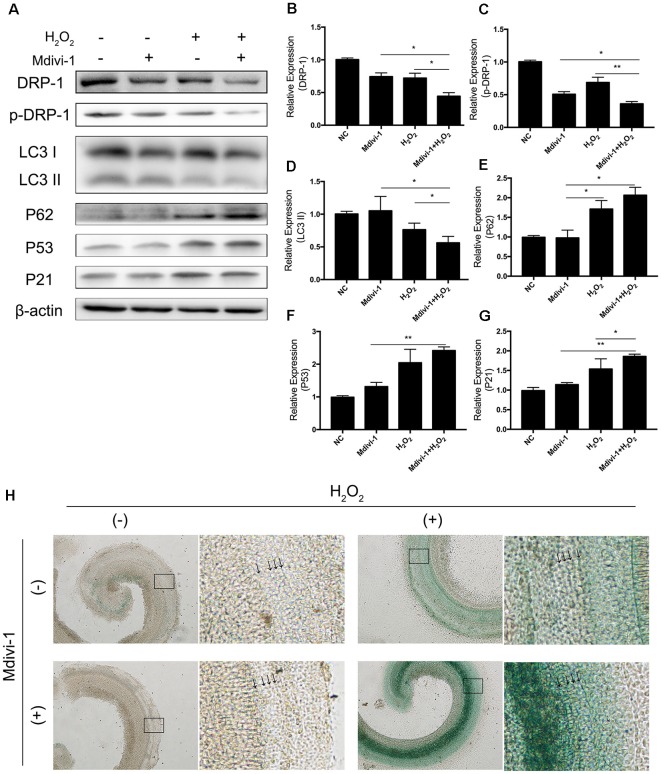
Mdivi-1 exacerbated senescence in H_2_O_2_-treated cochlea by inhibition of mitophagy. **(A)** Representative Western Blot analysis using antibodies against DRP-1, p-DRP-1, LC3 I/II, P62, P53, and P21 to assess mitophagy level and senescence in cochlear explants treated with H_2_O_2_ and DRP-1 inhibitor Mdivi-1. **(B,C)** Relative expression of DRP-1 and p-DPR-1. Mdivi-1 inhibited DRP-1 expression and phosphorylation. **(D,E)** Relative expression of LC3 II and P62. Mdivi-1 suppressed the mitophagy level in H_2_O_2_-treated cochlea. **(F,G)** Relative expression of P53 and P21. Mdivi-1 aggravated H_2_O_2_-induced senescence in cochlear explants. **(H)** Representative scanned images of the cochlear surface with β-gal staining of cochlea. With Mdivi-1, cochlea had a darker greenish color under H_2_O_2_ treatment. Black arrows indicate OHC and IHC. *p < 0.05, **p < 0.01.

Mitochondrial-division inhibitor Mdivi-1 inhibits mitochondrial fission by deactivation of DRP-1. To determine whether DRP-1 has an effect on cochlear senescence, we assessed the mitophagy function and senescent phenotype in the cochlear explants. We evaluated mitophagy in cochlear explants with pre-treatment with 50 μM of Mdivi-1 for 4 h before H_2_O_2_ treatment. Mdivi-1 pre-treatment de-activated DRP-1 and significantly down-regulated the mitophagy level under oxidative stress (Figures 5B,C). The LC3 II and P62 levels were evaluated to assess autophagy flux and mitophagy (Figures 5D,E). Western Blotting of protein extracts of H_2_O_2_-exposed cochlea pre-treated with Mdivi-1 revealed damaged mitophagy by down-regulation of LC3 II (*p* = 0.0243) and upregulation of P62 (*p* = 0.0022, Figures 5A–E). H_2_O_2_-induced cellular senescence was exacerbated by DRP-1 silencing. To further investigate the role of DRP-1 in aged cochlea, we assessed senescence-associated P53, P21 (Figures 5F,G), and β-gal stain in the cochlear explants. Mdivi-1 pre-treatment did not solely induce senescence in cochlea but exacerbated the senescent damage induced by H_2_O_2_ (Figure 5H). These results indicated that H_2_O_2_ inhibited mitophagy and could further induce senescence in cochlea.

### Mdivi-1 Inhibited Mitophagy and Induced Hearing Loss in Aged Mice

**Figure 6:**
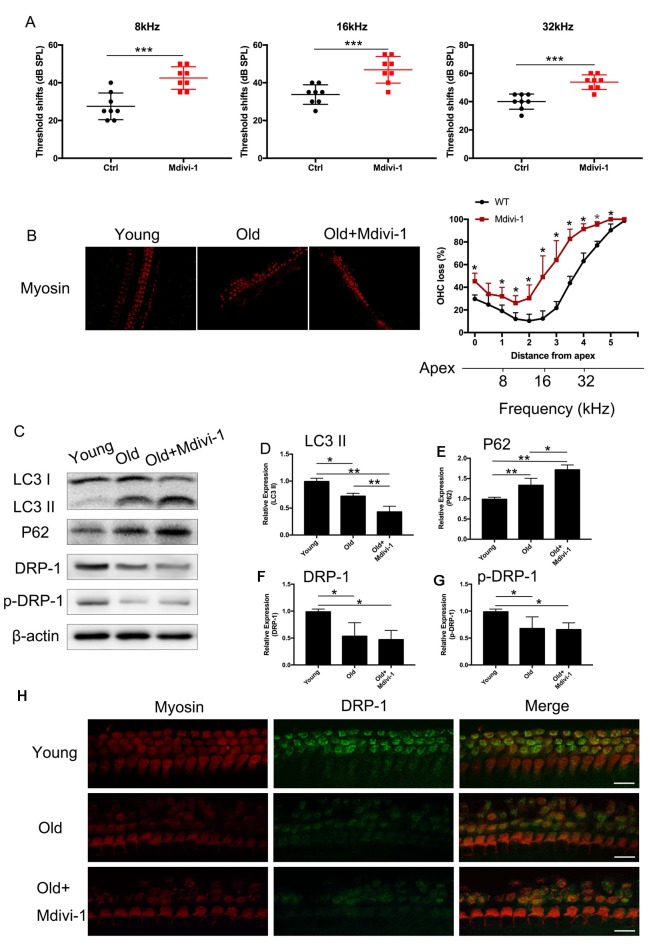
Administration of Mdivi-1 in C57BL/6 mice resulted in greater hearing loss and hair cell loss. **(A)** Auditory brainstem response (ABR) threshold shifts in C57BL/6 mice. One-month-old mice were considered as young control. There was a significant hearing threshold shift in 12-month-old Mdivi-1 IP mice compared to the control mice. Mdivi-1 IP administration resulted in greater shifts compared to in aged mice. **(B)** Hair cell count in the basilar membrane of young, old control, and Mdivi-1 IP mice. **(C–H)** Mitophagy level and DRP-1 expression assessment by LC3 II, P62, DRP-1, and p-DRP-1, comparing the cochlea of young, old control, and Mdivi-1 IP mice. **(H)** Representative image of immunofluorescence staining of DRP-1 in the cochlea of young, old, and old with Mdivi-1 administration mice. Significant down expression of DRP-1 in old-mouse cochlea compared to the young control mice. *p < 0.05, **p < 0.01, ***p < 0.001, scale bar = 100 μm.

C57BL/6 mice are known for their early-onset hearing loss and are often used as AHL models. To examine the hearing function of C57BL/6 mice, we performed ABR tests on aged and young C57BL/6 mice. We used 1-month-old mice as normal control, where hearing function had fully developed but no hearing loss had occurred. We evaluated the hearing threshold of the normal control group and a Mdivi-1 treatment group of 12 months old, by which time the C57BL/6 mice had developed hearing loss. The Mdivi-1 group was given Mdivi-1 *via* intraperitoneal administration beginning at the 8th month, before the C57BL/6 mice had developed hearing loss. The Mdivi-1 group developed greater threshold shifts at 8, 16, and 32 kHz than control mice and also exhibited greater hair cell loss (Figures 6A,B). We evaluated mitophagy using Western Blotting analysis, and the results revealed differences between aged mice and Mdivi-1-treated mice. There was a decrease of LC3 II (*p* = 0.013) and an increase of P62 (*p* = 0.0266, Figures 6C–E). Also, we found a decline of expression of DRP-1 and p-DRP-1 in aged cochlea (Figures 6F–H). However, there was no significant difference between aged mice and Mdivi-1-treated mice. The results indicated that mitophagy might be an effective way of hair cells survival under age stress and that inhibition of DRP-1 may block mitophagy and induce AHL.

## Discussion

Mitochondria form the cellular energy factory that synthesizes and provides ATP. However, dysfunctional mitochondria produce less ATP with more ROS and are more likely to induce apoptosis, necrosis, and senescence. Thus, there is a complicated and delicate mechanism of mitochondrial quality control to maintain mitochondrial function; this consists of mitochondrial fission and fusion, mitochondrial biogenesis, and mitophagy. Mitophagy is a process of selective cellular defense. Through this process, damaged mitochondrial matrices can be separated and eliminated to maintain mitochondrial function and homeostasis and alleviate cellular injury. The inhibition of mitophagy results in mitochondrial dysfunction and cellular senescence (García-Prat et al., 2016). On the other hand, an increase in mitophagy repairs cellular injury and delays the process of senescence (Dalle Pezze et al., 2014; Manzella et al., 2018).

Mitochondrial fission and fusion depend on a series of GTPases (van der Bliek et al., 2013). DRP-1 is a dynamic-related GTP-binding protein. It is key in controlling mitochondrial fission and is reported to initiate mitophagy (Lemasters, 2005). DRP-1 induces mitophagy to attenuate oxidative damage in other organs like the spinal cord and neurons. Oxidative stress damage also occurs and results in AHL. Here, we investigate whether DRP-1-induced mitophagy plays a role in the process of AHL.

We first established a cellular senescence model of HEI-OC1 cells and studied the expression of DRP-1 and mitophagy levels. We found a decline of DRP-1 expression and mitophagy level along with mitochondrial dysfunction. These results indicated that there was a correlation between DRP-1 and mitochondrial dysfunction in senescent HEI-OC1 cells. We hypothesized that mitochondrial dysfunction was induced by a disturbance in mitophagy. Our previous study revealed that rapamycin can preserve mitochondrial function by initiating autophagy (Pang et al., 2017). We found that in cellular senescence, the disturbed autophagy flux assessed by the LC3 II and P62 proteins confirmed our hypothesis. Cells treated with rapamycin displayed a normal level of ATP but a decreased level of mtDNA, indicating that rapamycin induced autophagy and preserved mitochondrial function. However, with oxidative stress, low ATP content and a lower autophagy level were found in senescent cells. The above results suggested that, in HEI-OC1 cells, oxidative stress suppressed autophagy, which might also suppress mitophagy and induce mitochondrial dysfunction. Other studies have demonstrated that DRP-1-induced mitophagy rescued mitochondrial function. These studies were related to oxidative stress in cardiac aging (Fernández et al., 2018), PD (Martinez et al., 2018), and early-stage AD (Wang et al., 2019). Our research confirmed that the initiation of mitophagy depends on DRP-1 and that DRP-1-induced mitophagy is key in the regulation of oxidative damage in hair cells.

We further investigated DRP-1 and mitochondrial function. We evaluated mitochondrial membrane potential, ATP content, and mtDNA in DRP-1 overexpressing and silencing HEI-OC1 cells. Mitochondrial membrane potential displayed no differences between DRP-1 overexpressing and silencing cells. However, in cells in which senescence had been induced by oxidative stress, we found more injured mitochondria with low membrane potential in DRP-1 silencing cells but relatively less in DRP-1 overexpressing cells. The tendency was the same in ATP content. We defined mitochondrial dysfunction as poor membrane potential and low ATP content. mtDNA, on the other hand, would represent the amount of mitochondria. A low amount of mitochondria, assessed by mtDNA, was found in H_2_O_2_ treatment and rapamycin treatment. In DRP-1 overexpressing cells, autophagy flux was initiated, while ATP content and membrane potential remained normal. However, mtDNA was mildly decreased in DRP-1 overexpressing cells, suggesting that fewer mitochondria would produce more ATP. In DRP-1 silencing cells treated with H_2_O_2_, the autophagy flux was disturbed, and mitochondrial dysfunction was displayed. We suggested that failure to eliminate dysfunctional mitochondria was the reason why mtDNA was at a normal level. Also, there were fewer merging fluorescent puncta of mitochondria (red) and lysosome (green). The expression of the autophagy-related proteins LC3 II and P62 decreased. The results may suggest that DRP-1 is the key to regulating mitophagy and mitochondrial function. As cellular senescence developed, the decline of DRP-1 expression resulted in decreased mitophagy levels and an accumulation of dysfunctional mitochondria. We further investigated DRP-1 expression and senescence in cochlear explants. In senescent cochlear explants, DRP-1 expression and mitophagy levels decreased along with impaired mitochondrial function. These results are similar to those from the cellular experiment. Therefore, we hypothesized that age-related decline in DRP-1 repressed mitophagy and there was an accumulation of impaired mitochondria, which caused cochlear senescence.

In aged C57BL/6 mice as AHL models, we identified the downregulation of DRP-1 expression and mitophagy by evaluating the protein expressions of DRP-1, LC3 II, and P62. DRP-1 and mitophagy were downregulated in aged mice and senescent cochlea. Impaired mitochondria accumulated during aging due to a gradual decline in mitophagy and due to mitochondrial dysfunction. Our previous study revealed that initiating autophagy with rapamycin could notably improve hearing conditions in aged mice compared to AHL mice. In our current study, we applied Mdivi-1, an DRP-1 inhibitor, to C57BL/6 mice. These mice developed even more severe hearing loss than those who went without Mdivi-1. All of the above implied that the inhibition of DRP-1 expression and function resulted in decreased levels of mitophagy and further AHL development. It is possible that there are still unknown mechanisms that alter mitochondrial function (resulting in senescence) that need further study. We propose that DRP-1 could be the center of mitochondrial quality control and could be a key factor in AHL.

**Figure 7:**
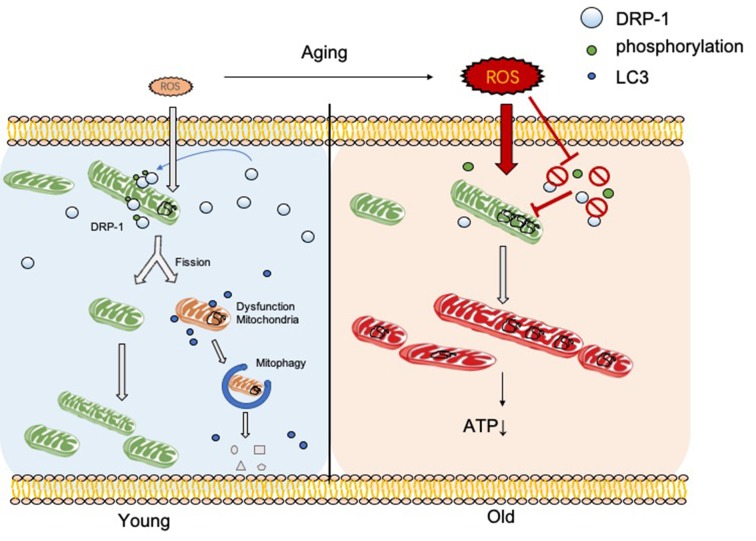
DRP-1 induces separation of damaged mitochondrial matrix before initiation of mitophagy in young cells. With aging, reactive oxygen species (ROS) and oxidative stress increase and inhibit DRP-1 function. Oxidatively damaged mitochondria accumulate and result in a dysfunctional ATP metabolism and thus cellular senescence.

In this research, we discovered the relationship between DRP-1 and hair cell senescence (Figure 7). We revealed the role of DRP-1 in AHL and examined the effects of inhibition of DRP-1 in hearing. However, there are still questions that remain to be answered in our study. One question regards mitochondrial morphology and mitochondrial function. According to some research, a giant mitochondrial network created through mitochondrial fusion can preserve damaged mitochondria to maintain their function. The dynamic morphology of mitochondria mismatches with mitochondrial function, making this unconvincing. Second, the exact mechanism that controls how oxidative stress suppresses DRP-1 expression and phosphorylation remains unknown. Research has implied that the phosphorylation of DRP-1 may affect the ROS-AMPK pathway (Li et al., 2017), which is one of the critical pathways in oxidative regulation and may be an essential target in the study of senescence and AHL. Third, our study would be more convincing if it were to show whether DRP-1 delays the development of AHL in transgenic mice. Lastly, we revealed that DRP-1 participated in the development of AHL and considered it as a critical part of AHL. We hope to uncover the mechanisms of senescence and AHL by studying DRP-1 and related to DRP-1 that could prevent, delay, or even cure AHL in the future.

## Data Availability Statement

All datasets generated for this study are included in the article.

## Ethics Statement

The animal study was reviewed and approved by Sun Yat-sen University.

## Author Contributions

HL and HX: contributed equally to this work. HL: conception and design, designed and performed the experiments, data analysis and interpretation, and manuscript writing. YZ and HX: conception and design, manuscript writing, final approval of manuscript. ZS, JP, LL, HZ, BJ, and WZ: assembly of data and data analysis.

## Conflict of Interest

The authors declare that the research was conducted in the absence of any commercial or financial relationships that could be construed as a potential conflict of interest.
